# Renal macro- and microcirculation autoregulatory capacity during early sepsis and norepinephrine infusion in rats

**DOI:** 10.1186/cc12818

**Published:** 2013-07-12

**Authors:** Mélanie Burban, Jean-François Hamel, Maher Tabka, Mathilde Renou de La Bourdonnaye, Agnès Duveau, Alain Mercat, Paul Calès, Pierre Asfar, Nicolas Lerolle

**Affiliations:** 1 Laboratoire HIFIH, UPRES EA 3859, PRES l'UNAM, IFR 132, Université Angers, 49000 Angers, France; 2 Centre de recherche clinique, Centre hospitalier Universitaire Angers, 49933 Angers, France; 3 Département de Réanimation Médicale et de Médecine Hyperbare, Centre Hospitalier Universitaire Angers, 49933 Angers, France; 4 CNRS UMR 6214, INSERM U1083, Université d'Angers, Angers, France

**Keywords:** Sepsis, animal, acute renal failure, hemodynamic, experimental, norepinephrine

## Abstract

**Introduction:**

The relationships between systemic hemodynamics and renal blood flow and renal microcirculation are poorly known in sepsis. Norepinephrine (NE) infusion may add another level of complexity.

**Methods:**

Ventilated and anesthetized rats were submitted to various mean arterial pressure (MAP) steps by blood removal, in presence and absence of sepsis and/or NE. Renal blood flow (RBF) and blood velocity (Vm) in renal cortical capillaries (using Sidestream Dark Field Imaging) were measured. Data were analyzed using linear mixed models enabling us to display the effects of both the considered explanatory variables and their interactions.

**Results:**

Positive correlations were found between MAP and RBF. Sepsis had no independent impact on RBF whereas norepinephrine decreased RBF, regardless of the presence of sepsis. The relationship between MAP and RBF was weaker above a MAP of 100 mmHg as opposed to below 100 mmHg, with RBF displaying a relative "plateau" above this threshold. Sepsis and NE impacted carotid blood flow (CBF) differently compared to RBF, demonstrating organ specificity. A positive relationship was observed between MAP and Vm. Sepsis increased Vm while nNE decreased Vm irrespective of MAP. Sepsis was associated with an increase in serum creatinine determined at the end of the experiments, which was prevented by NE infusion.

**Conclusion:**

In our model, sepsis at an early phase did not impact RBF over a large range of MAP. NE elicited a renal vasoconstrictive effect. Autoregulation of RBF appeared conserved in sepsis. Conversely, sepsis was associated with "hypervelocity" of blood flow in cortical peritubular capillaries reversed by NE infusion.

## Introduction

Acute kidney injury (AKI) is a major complication of sepsis and is associated with increased mortality [1]. Renal vascular dysfunction has emerged as a major component of AKI [2-4]. First, on a 'macrovascular' level, renal response to vasoconstrictor or vasodilator substances at the arterial and arteriolar level is altered in the presence of sepsis toward an increased vasopressive response [5]. Second, on a 'microvascular' level, several recent lines of evidence have shown that abnormalities in cortical peritubular capillary flow occur very early in sepsis [6-8].

In animal models of sepsis, renal blood flow (RBF) is preserved provided that cardiac output is maintained with vascular fluid loading [2]. Nonetheless, the abovementioned alterations in vasoreactivity may render the kidneys unable to protect themselves against additional ischemic insults during changes in systemic perfusion pressure: for instance, mean arterial pressure (MAP) variations may result in a rapid drop in RBF and/or glomerular filtration rate (GFR). Indeed, suboptimal perfusion pressure is common in patients with septic shock and has been shown to be associated with renal dysfunction [9]. Most animal studies on RBF in sepsis have used either no fluid loading leading to profound hemodynamic abnormalities or massive fluid resuscitation leading to 'overcorrection' of cardiac output and arterial pressure, such that the impact of variation of blood pressure over a range relevant to the human setting remains unknown [2].

Aside from RBF, the relationship between systemic hemodynamics and microcirculation remains a matter of debate. In experiments performed in various organs, the link between systemic hemodynamics and microcirculation has been relatively vague although the latter was found to be affected by cardiac output and arterial pressure when these were critically altered [10]. Most observations in kidneys, showing a microcirculatory defect in sepsis conditions, have been conducted in non-resuscitated animals such that cardiac output, and hence RBF, may have been substantially diminished [7,8].

As a result, the manner in which frequent MAP variations in sepsis impact both RBF and microcirculation is unknown. Moreover, septic shock patients require vasoconstrictor infusion, mostly norepinephrine (NE), in addition to fluid loading, to maintain blood pressure which may add another level of complexity to the vascular abnormalities observed in sepsis [11].

Thus, in order to explore the relationships between variations in MAP over a clinically relevant range and renal macro- and cortical microcirculations, we conducted a study on a rat model of sepsis with and without NE infusion. Renal cortical peritubular capillary blood flow was monitored using Sidestream Dark Field (SDF) imaging [12]. Our main objectives were: (1) to assess the existence of an autoregulation phenomenon between MAP and RBF in sepsis and whether it is impacted by NE; and (2) to evaluate if changes in MAP impact cortical microcirculation in a manner similar to RBF in septic condition, in presence/absence of NE.

## Materials and methods

### Animal preparation

Adult male Wistar rats, weighing 250 ± 20 g (aged approximately 8 weeks), were housed under 12-h light/dark cycles in the animal facility of the University of Angers (France). All experiments were performed in compliance with the European legislation on the use of laboratory animals and have been approved by the Comité Régional d'Ethique pour l'Expérimentation Animale (Regional Animal Ethics Committee) of Nantes, France (approval number: 2011.19).

The rats were anesthetized with intraperitoneal pentobarbital (50 mg/kg, Trapanal^®^, Nycomed, Germany) associated with local anesthesia for each incision (Lidocaïne^®^1% Astra Zenecca) and placed on a homeothermic blanket system to maintain rectal temperature between 36.8°C and 37.8°C. A tracheotomy was performed and animals were mechanically ventilated (Harvard Rodent 683 ventilator, Harvard Instruments, South Natick, MA, USA) with supplemental oxygen in order to maintain PaO_2 _around 100 mmHg and PCO_2 _at 35-45 mmHg. The left carotid artery and renal artery were exposed, and 2.0 mm transit-time ultrasound flow probes (Transonic Systems Inc., Ithaca, NY, USA) were placed to continuously measure carotid blood flow (CBF) and RBF. The left femoral artery was cannulated and connected to a transducer (Edwards LifesciencesTM, Irvine, CA, USA) allowing continuous measurements of MAP and heart rate (HR). The homolateral femoral vein was cannulated and maintenance of fluid was performed with a perfusion of 1.2 mL/h of 0.9% NaCl. Arterial blood gases (Blood gas analyzer, Osmetech Opti™ CCA, Pantin, France) were performed after animal preparation in order to adjust for mechanical ventilation. The left kidney was exposed and a SDF imaging camera (MicroScan™; MicroVisionMedical, Amsterdam, The Netherlands) was positioned on the surface. Special attention was paid to apply the lowest pressure on the kidney surface with the camera.

### Experimental protocol

This prospective experimental study randomly allocated rats into four groups (*n *= 10 per group):

- In the CT (control) group, after a baseline step (at a typical MAP of 130 mmHg in Wistar rats), MAP was lowered by blood removal via the arterial catheter to reach three successive predefined MAP steps lasting 10 min each: 100 mmHg, 70 mmHg, and 40 mmHg. Shed blood (stored in a syringe containing heparin 200 UI) was then reinfused. During the MAP steps, small amounts of blood were occasionally withdrawn or reinfused to maintain MAP at the predefined levels.

- In the Sepsis group, cecal ligation and puncture was performed immediately after animal preparation. In brief, under aseptic conditions, a 3-cm midline laparotomy was performed. The cecum was partially ligated and perforated with an 18-gauge needle and gently squeezed to extrude a small amount of feces. The cecum was then returned to the peritoneal cavity and the laparotomy was closed with 4.0 silk sutures. MAP was then allowed to decrease spontaneously to 90 mmHg at which point gelatin (Gelofusine 4%, B. Braun Medical, Boulogne, France) was perfused to restore a MAP of 130 mmHg, which was considered as the baseline step in this group. The experimental protocol described for the CT group was then applied.

- In the CT+NE (control+NE) group, continuous intravenous administration of NE was titrated after animal preparation to obtain a MAP of 160 mmHg (baseline step). The experimental protocol described for the CT group was then performed with a first step at MAP 130 mmHg.

- In the Sepsis+NE group, the protocol described for the Sepsis group was applied, NE being started to obtain a MAP step of 160 mmHg (baseline step). This was performed immediately after gelatin infusion, which had enabled to restore a MAP of 130 mmHg after the initial MAP drop. The experimental protocol was then applied as above.

At each step (including baseline), three sets of simultaneous measurements were performed for MAP, HR, CBF, RBF, and cortical microcirculatory velocities (Vm, see below). At the end of the experiments, animals were sacrificed with a lethal dose of pentobarbital and blood was drawn for creatinine measurements (CreatininasePeroxydase, C16000 Abbott, IL, USA).

### Microcirculation imaging and peritubular blood velocity measurements

Video sequences of 10-s duration were obtained with a SDF camera for every set of measurements. The camera was slightly displaced between each sequence to allow observation of different regions in the superficial area of the cortex. Digital sequences were stored and analyzed off-line with specifically-designed software (AVA 3.0, MicroVisionMedical, Amsterdam, The Netherlands). Video analyses were performed blinded to both group and step. After image stabilization and averaging, displacements of blood cells in cortical peritubular capillaries were observed (see Additional file 1, Video S1). White blood cell displacement through the capillaries creates an observable artifact among red blood cells; the velocity of this artifact can be measured using time-frame analysis, as previously reported. In capillaries (blood vessel diameter <10 μm), blood cell velocity can be considered as blood velocity [13]. For each video sequence, blood velocities were measured in five to 10 different blood vessels and averaged. In a pre-study, intra-observer reproducibility testing for blood velocity measurements over a video sequence displayed good reliability of measurements (inter-class correlation 94%; 95% CI 92-99%). Inter-observer reproducibility testing also displayed good agreement between operators (inter-class correlation 90%; 95% CI 88-97%).

### Statistical analyses

Data are presented as mean ± standard deviation, and *n *(%) as appropriate. Quantitative data were compared with the Kruskall-Wallis test or Wilcoxon test as appropriate, and qualitative data with the Fisher exact test.

Different measurements carried out on a same rat are necessarily correlated, whether made for the same blood pressure level or not. As standard statistical models (such as linear models) are based on the observations independence assumption, their use was deemed inappropriate. Mixed models are particularly suitable for repeated measures and for modeling correlations between measurements and were thus used herein [14]. In a first model, we assessed the determinants of RBF among MAP, Sepsis, NE infusion and tested whether the relationship between MAP and RBF was different above and below a MAP of 100 mmHg, by testing the interaction between MAP and this threshold (hereafter referred as T100 mmHg). In a second model, we assessed the determinants of CBF among the same parameters as above (MAP, Sepsis, NE infusion, and interaction between MAP and T100 mmHg). In a third model, we assessed the determinants of Vm among MAP, Sepsis, and NE infusion. These models allowed to display the effects of both the considered explanatory variables and their interactions (considered as fixed effects) while simultaneously modeling random effects. Random effects describe the difference between actual values of the studied parameters as measured during the experiments and theoretical values predicted by the model when entering the fixed effects. The random effects were broken down into inter-animal variability (describing how different animals respond differently to the same stimuli), and intra-animal variability (describing how a same animal responds differently to the same stimuli, as estimated from the three measurements at each MAP step). Intra-animal variabilities were estimated depending on the septic and NE status of the animals. The models were constructed by minimizing Akaïke criteria. Only significant interactions between variables were retained and presented. The models presented were validated by verifying the normality and homoscedasticity assumptions of the residuals. The significance of covariates was tested by the use of Wald tests. All tests were performed with a type I error set at 0.05. Analyses were performed using Stata 11.0 (StataCorp LP, TX, USA).

## Results

### Hemodynamic measurements: MAP steps, heart rate, NE infusion

**Figure 1:**
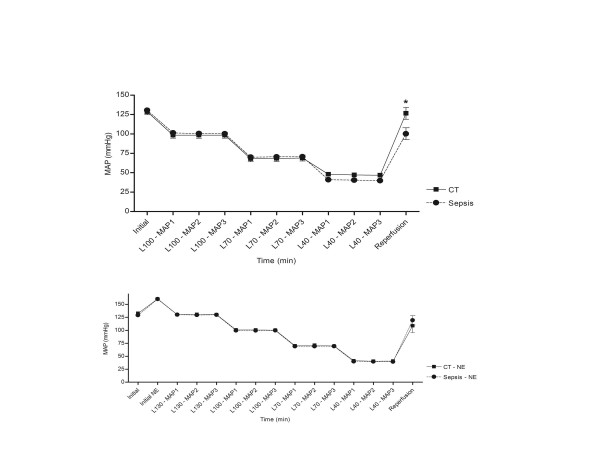
**Graphical representation of mean arterial pressure (MAP) across the different experimental groups**. Baseline (see materials and methods) measurements were considered as time = 0. CT: control; L: level; NE: norepinephrine.

The MAP targets were achieved successfully in all four experimental groups (Figure 1). The volume of Gelofusine required to obtain a MAP baseline step of 130 mmHg in septic animals was 4.2 ± 4 mL in the Septic group and 3.6 ± 2 mL in the Sepsis+NE group (before NE was initiated). After reperfusion of shed blood, MAP was significantly lower in comparison to baseline in all groups except CT (CT 127 ± 26 *vs*. 129 ± 5, *P *= 0.8; Sepsis 100 ± 35 *vs*.130 ± 4, *P *= 0.002; CT+NE 109 ± 41 *vs*. 161 ± 7, *P *= 0.01; Sepsis+NE 119 ± 30 *vs*. 160 ± 8 mmHg, *P *= 0.004). MAP had no significant effect on HR whereas sepsis and NE infusion were associated with a significant increase in HR (data of the linear mixed model not shown; for example, HR at baseline: CT 324 ± 63; Sepsis 427 ± 51; CT+NE 342 ± 45; Sepsis+NE 371 ± 66 bpm). The NE infusion rate required to obtain a MAP of 160 mmHg at baseline was 6.2 ± 3.8 μg/kg/min in CT+NE animals and 24.05 ± 5.9 μg/kg/min in Sepsis+NE animals (*P *= 0.0002). Duration of the experiment from end of preparation to reperfusion was approximately 70 min in CT and 90 min in CT+NE. This duration increased to 300 ± 69 min and 359 ± 97 min (*P *= 0.005) in Sepsis and Sepsis+NE, respectively. Animal weight did not differ between the various groups (CT 260 ± 30 g, Sepsis 246 ± 20 g, CT+NE 247 ± 23 g, Sepsis+NE 266 ± 20 g, *P *= 0.45).

### Macrocirculatory impact of MAP steps: renal and carotid blood flow

**Figure 2:**
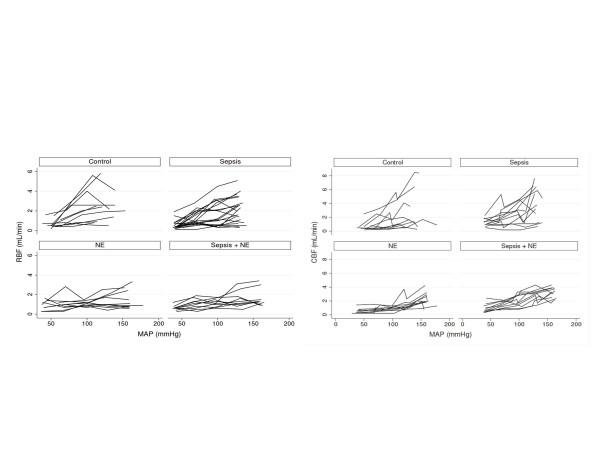
**RBF (left kidney), CBF (left carotid), and Vm according to MAP in the different experimental groups**. Only the first of the three sets of measurements for each MAP step are shown. However, the statistical analyses presented in the article take into account all of the measurements. CBF: cerebral blood flow; MAP: mean arterial pressure; RBF: renal blood flow; Vm: microcirculatory velocity.

RBF and CBF values according to measured MAP are shown in Figure 2, while numerical data are presented in Additional file 2 (Table S1). Results of the mixed linear regression models disclosing the effect of MAP, sepsis, and NE on RBF and on CBF are shown in Table 1. Coefficients presented here apply to a first order equation: for example, the variation in RBF when MAP increases by 1 mmHg is 1 × 0.05 mL/min. Identically, for NE infusion, RBF variation in presence/absence of NE is -0.9 × (1 in presence of NE, 0 in absence of NE) mL/min.

**Table 1:** Linear mixed models for renal blood flow (RBF) and cerebral blood flow (CBF).

RBF	Coefficient	95% confidence interval	*P*
*Fixed effects*			
MAP (mmHg)	0.05	0.01; 0.15	<0.001
NE infusion	-0.90	-1.24; -0.57	<0.001
Sepsis	-0.26	-0.72; 0.2	0.3
Interaction MAP×T100 mmHg^a^	-0.01	-0.01; -0.001	0.001
*Random effects*			
Interindividual standard deviation	0.78	0.61; 0.98	
Intraindividual standard deviation			
Control	1.45	1.27; 1.7	<0.0001^a^
Sepsis	0.9	0.81; 1	
NE infusion	0.53	0.46; 0.6	
NE+Sepsis	0.39	0.35; 0.44	

**CBF**	**Coefficient**	**95% confidence interval**	***P***

*Fixed effects*			
MAP (mmHg)	0.02	0.02; 0.03	<0.001
NE infusion	1.83	1.24; 2.40	<0.001
Sepsis	0.76	0.15; 1.37	0.01
Interaction MAP × NE^c^	-0.02	-0.02; -0.01	<0.001
Interaction MAP×T100 mmHg^a^	0.013	0.01; 0.002	<0.001
			
*Random effects*			
Interindividual standard deviation	0.99	0.77; 1.27	
Intraindividual standard deviation			
Control	1.01	0.88; 1.16	<0.001^a^
Sepsis	0.91	0.8; 1.03	
NE infusion	0.38	0.33; 0.43	
NE+Sepsis	0.62	0.55 ; 0.7	

^a^Interaction MAP × threshold 100 mmHg.

^b^For between-groups comparison.

^c^Interaction MAP × NE infusion.

MAP: mean arterial pressure; NE: norepinephrine.

Thus, regarding RBF, the model shows that MAP significantly increased RBF, regardless of NE infusion and septic status (no significant interaction between MAP and NE or between MAP and Sepsis detected by the model). The MAP-dependent increase in RBF varied according to the 100 mmHg MAP threshold: when MAP was >100 mmHg, the increase in RBF in response to a rise in MAP was lower than that observed when MAP was <100 mmHg: when MAP was >100 mmHg, increasing blood pressure by 1 mmHg increased RBF by 0.04 mL/min (0.05 minus 0.01: effect of MAP plus effect of interaction between MAP and 100 mmHg threshold on RBF), whereas when MAP was <100 mmHG, increasing blood pressure by 1 mmHg increased RBF by 0.05 mL/min (single effect of arterial pressure on RBF; the effect of interaction is by design equal to zero as MAP >100). Of note, this threshold was chosen *a priori *as it is considered as the usual 'autoregulation' threshold of the relationship between MAP and RBF in rats [15]. NE infusion significantly decreased RBF, irrespective of septic status and MAP measurement (no interaction between NE and MAP and between NE and Sepsis was detected). Sepsis had no significant impact on RBF. Intra-animal RBF variability differed significantly among the different groups. Sepsis and NE decreased variability, which was lowest in Sepsis+NE animals. In other words, in the presence of NE and sepsis, the change in RBF in a given animal was more predictable using the fixed effects included in the model (particularly the MAP variation) than in the absence of these events.

Regarding CBF, MAP significantly increased CBF, regardless of septic status. The MAP-dependent increase in CBF varied according to the 100 mmHg MAP threshold and NE infusion. When MAP was >100 mmHg, the increase in CBF was greater (as opposed to that observed for RBF) in response to a rise in MAP than when MAP was <100 mmHg (positive interaction between the MAP effect and the 100 mmHg threshold effect). The increase in CBF in response to an increase in MAP was lower than when rats were perfused with NE (negative interaction between the MAP effect and the NE infusion effect). However, when rats were perfused with NE, CBF was increased regardless of septic status and MAP measurement. CBF was also increased in presence of sepsis, regardless of NE infusion status and MAP measurement. Intra-animal CBF variability was reduced in the presence of NE and to a lower extent in Sepsis+NE animals.

Beyond the analysis of the interaction between the 100 mmHg threshold and MAP, we compared the variations in RBF and CBF when MAP was decreased from 130 mmHg to 100 mmHg among the different groups (Table 2). No significant difference in RBF variations was observed between control, Sepsis, NE, and Sepsis+NE groups when MAP was lowered to this level. This observation could indicate that the slope of the MAP/RBF relationship may be similar across all groups in this range of MAP. In contrast, CBF variations seemingly differed among the various groups in that the decrease in CBF appeared to be lower in the presence of NE infusion.

**Table 2:** Variation in renal and carotid blood flow for a 130-to-100 mmHg mean arterial pressure drop.

	Δ Renal blood flow		Δ Carotid blood flow	
	Value (mL/min)	*P*	Value (mL/min)	*P*
Control	-0.66 ± 1.35	0.92	-1.53 ± 1.20	<0.01
Sepsis	-0.59 ± 0.93		-1.60 ± 1.35	
NE infusion	-0.45 ± 0.70		-0.54 ± 0.38	
NE+Sepsis	-0.70 ± 0.55		-0.68 ± 0.52	

NE: norepinephrine.

### Renal cortical peritubular microcirculation

**Figure 3:**
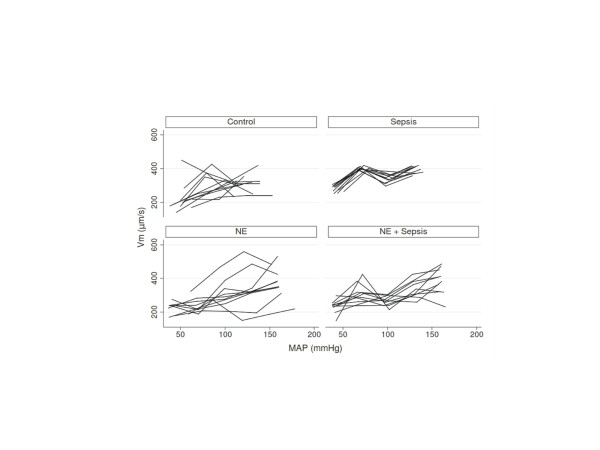
**Vm according to MAP in the different experimental groups**. Only the first of the three sets of measurements for each MAP step are shown. However, the statistical analyses presented in the article take into account all of the measurements. MAP: mean arterial pressure; Vm: microcirculatory velocity.

Figure 3 shows Vm profiles according to measured MAP (numerical data provided in Additional file 2, Table S1) while Table 3 summarizes the linear mixed model for Vm. A significant positive relationship was observed between MAP and Vm. Moreover, NE infusion decreased blood velocity whereas sepsis enhanced this parameter. No interaction was detected between these different effects, that is, the effects described above were observed regardless of the other tested parameters. Intra-animal variability for Vm was significantly different between groups, being lower in Sepsis. Of note, when RBF was considered in lieu of MAP in a linear mixed model, similar fixed and random effects were observed (data not shown) such that for a given RBF, Vm was reduced by NE while enhanced by sepsis.

**Table 3:** Linear mixed model for microcirculatory velocity (Vm) in peritubular cortical capillaries.

Vm	Coefficient	95% confidence interval	*P*
*Fixed effects*			
MAP (mmHg)	1.08	0.94; 1.23	<0.001
NE infusion	-77.3	-96.6; -58	<0.001
Presence of sepsis	48.1	22.4; 73.7	<0.001
			
*Random effects*			
Interindividual standard deviation	39.9	30.1; 52.8	
Intraindividual standard deviation			
Control	57.5	49.4; 66.9	<0.001^a^
Sepsis	49.8	43.4; 57.2	
NE infusion	53.9	47.3; 61.4	
NE+Sepsis	59.7	52.9; 67.4	

^a^For between groups comparison.

MAP: mean arterial pressure; NE: norepinephrine.

### Renal function

Serum creatinine measurements performed at the end of the experiments were significantly different among the different groups, notably sepsis induced an increase in serum creatinine, which was prevented in the presence of NE (CT 37 ± 2, CT+NE 40 ± 6, Sepsis 68 ± 12, Sepsis+NE 41 ± 4 μmol/L, *P *= 0.0001).

## Discussion

The present animal study was aimed at delineating the 'macro' and 'micro' hemodynamic changes over a broad range of mean arterial pressures in septic condition with or without NE infusion. The original design of our study with controlled variations in MAP allowed entering MAP, NE and sepsis as independent variables and assess RBF or CBFand cortical peritubular capillary blood velocity as dependent variables. Mixed models are particularly suitable for repeated measures and for modeling correlations between measurements and were hence used in the present setting. As sepsis is characterized by altered variability of clinical and biological signals, assessing variability of measurements was considered [16,17]. Consequently, we paid special attention to display all collected data in figures.

As expected, positive correlations were found between MAP and RBF as well as between MAP and CBF: namely, the higher the MAP, the higher the blood flow in these vessels. Sepsis did not appear to have an independent impact on RBF; conversely, NE decreased RBF. Indeed, in the present model, for any given MAP, RBF remained similar in the presence or absence of sepsis, but was lower in the presence of NE, thus providing evidence that NE displays a renal vasoconstrictive effect in this rat model. Of further importance, the relationship between MAP and RBF was weaker at MAP levels >100 mmHg in comparison to <100 mmHg. This is in accordance with an autoregulation phenomenon, although we did not observe a true RBF 'plateau'. More specifically, the variation in RBF when MAP varied from 100 to 130 mmHg did not differ in the presence or absence of sepsis or of NE, which may argue for the existence of this autoregulation phenomenon irrespective of the presence of sepsis or NE. Some of the observations pertaining to the kidney were organ specific since sepsis, NE and the MAP 100 mmHg threshold impacted CBF differently: both sepsis and NE increased CBF while the relationship between MAP and CBF was increased above 100 mmHg. Finally, a positive relationship was observed between MAP and renal cortical peritubular blood velocity (Vm) in that the higher the MAP, the higher the Vm. For a given MAP, sepsis increased Vm while NE decreased this velocity, such that NE tended to 'antagonize' the effect of sepsis on Vm. Finally, sepsis was associated with an increase in serum creatinine, which was prevented by NE.

RBF in sepsis has long been a subject of debate given that some models displayed decreased RBF while others showed either normal or elevated RBF. Langenberg et al. reconciled these conflicting data by demonstrating that the main parameter that predicted RBF in the various published animal models was the maintenance of cardiac output through fluid loading [2]. Herein, we confirm these findings, as previously observed, after restoring normal MAP through fluid loading, RBF did not differ in the presence/absence of sepsis. Furthermore, we extend these data by showing that the relationship between MAP and RBF is not altered and that the relative RBF 'plateau' observed above a MAP of 100 mmHg, usually referred to as 'autoregulation' phenomenon, appeared to be preserved in sepsis or in the case of NE infusion [15]. This last observation deserves to be emphasized since 'autoregulation' alteration is frequently cited as a contributing factor to renal failure [18]. The ability of the kidney to maintain RBF when MAP varies over a certain range is enabled both by the myogenic reflex and the tubuloglomerular feedback and is impacted by neuroendocrine kidney vasomotor regulation [19]. These effectors are dependent upon the integrity of several renal structures (that is, vascular, tubular) working in cooperation. It should be acknowledged that we only assessed the ensuing few hours after induction of sepsis, likely before renal damage may have sufficiently evolved to alter these structures. Indeed, several experimental data have shown that renal hemodynamics may be totally different at the initiation, maintenance, and recovery phases of AKI [16,20].

Cardiac output was not monitored in the present study. Inserting a Doppler probe around the thoracic aorta is very invasive in rats and we felt it would have added an unwanted form of insult to our rat model. Indeed, our model is very complex and sensitive to any supplemental form of aggression, especially in septic animals, and thus we had to carefully put into balance the relevance of any additional procedure and the risk that such procedure may destabilize the entire model. It should also be emphasized that the present study was essentially 'kidney centered' with the main objective to assess the existence or not of an autoregulation phenomenon, which is evaluated by the relationship between MAP and RBF. Thus, we chose to monitor RBF at 'controlled' levels of MAP, and not cardiac output, in order to provide a direct evaluation of this relationship. We believe that not measuring cardiac output could have been a problem in interpreting RBF had we observed a reduced RBF. Conversely, the absence of impact of sepsis on RBF indirectly indicates that cardiac output was not compromised, at least significantly, in our resuscitated model. In fact, absence of cardiac output measurements prevented us from determining whether renal and/or systemic resistances varied in a similar manner or not, that is, whether renal behavior is different from other vascular beds. A partial response to this answer was to monitor carotid blood flow, which is easy to access. Indeed, we observed that RBF and CBF did not vary identically.

Deciphering the direct renal effect of NE infusion has been especially difficult as this drug also impacts systemic hemodynamics, which in turn modifies renal circulation [18]. Indeed, most models showed that NE infusion was associated with an increased RBF together with an increased MAP and cardiac output [21]. The particular design of our study allowed to 'offset' the systemic effect of NE and to observe a direct vasoconstrictive effect of NE on renal vasculature. Influence of sepsis on the effect of NE on RBF adds another level of complexity to the already discrepant results reported in the literature. Some authors evidenced that the increase in RBF after NE infusion in septic animals was not completely explained by an increase in perfusion pressure indicating that, in this condition, NE may actually decrease renal vascular resistance [22]. Other studies showed a preservation of the pressor response to norepinephrine in the kidney, albeit with a diminished response in other vascular beds [5]. In the present study, we did not observe any modification of the renal response to NE incurred by sepsis. However, we should acknowledge the limitation of our model due to sample size, such that a small impact of sepsis on the effect of NE on renal vasculature cannot be ruled out.

Few data have been published to date on renal microcirculation changes in sepsis. To our knowledge, no report has used SDF imaging to assess kidney microcirculation until now. We observed that this technique allows an easy and direct observation of blood cell velocities in peritubular capillaries. In capillaries (blood vessel diameter <10 μm), blood cell velocity can be considered as blood velocity, such that we believe the parameter measured herein is a relevant index of renal cortical microperfusion [13]. It was observed that sepsis induces cortical peritubular blood cell hypervelocity, which was corrected by NE. Also noteworthy is that sepsis seems to impact cortical peritubular microcirculation and RBF differently in sepsis. Studies using intravital microscopy have previously been conducted on non-reanimated mice and rats by the group of Mayeux et al. [7,8,23]. These experiments showed a rapid decrease in the proportion of peritubular capillaries with continuous flow after induction of sepsis, which we did not observe in this study (data not shown). However, absence of fluid loading following sepsis induction in these particular experiments is likely to have resulted in a drastic drop in MAP, cardiac output, and RBF. Several publications have described the variation in cortical and sub-cortical oxygenation in septic condition in reanimated small animals using dual-wavelength oxygen phosphorimetry [6,24,25]. In these models, cortical oxygenation parameters were altered in septic animals and were only partially corrected by volume expansion allowing the normalization of RBF [24]. However, using oxygenation as a proxy for microcirculation may be debatable. Indeed, a recent study reported normal kidney oxygenation measured by blood oxygen level-dependent MRI in LPS-treated mice despite diminished RBF [26]. In this latter study, the authors evidenced profound mitochondrial alterations in the septic kidney, which could result in impaired oxygen utilization, such that oxygen tension may not be a good indicator of microvascular perfusion in sepsis.

Unfortunately, our findings do not allow to pinpoint the precise implications regarding acute kidney injury of peritubular capillary hypervelocity induced by sepsis and its correction by NE. Endothelial microvascular lesions have emerged as a component of kidney injury in several models, including sepsis or ischemia-reperfusion [4,27,28]. Peritubular capillary alterations have been the parameter most closely associated with renal failure among all vascular abnormalities described [28]. Peritubular capillaries immediately follow glomerular circulation, which determines the filtration capacity of the kidney. One hypothesis could be that sepsis elicits a diminished vascular tone of the glomerulus efferent artery resulting in both a hypervelocity in the ensuing capillaries and a drop in glomerular filtration pressure. NE may correct this vascular tone thus restoring normal capillary velocity and filtration pressure. To date, however, exploring glomerular circulation to confirm this hypothesis *in vivo *has been beyond the reach of current technical capabilities. Of note, whether velocity of blood cells is an accurate index of blood flow is difficult to establish. In addition to velocity, the diameter of blood vessels and the number of blood vessels perfused should be taken into account. Although we did not observe variations in capillary sizes (that is, no vascular ectasia) or the appearance of non-perfused vessels (data not shown), the precision of these latter observations do not allow us to extrapolate accurate blood flow measurements from our dataset. Only cortical peritubular capillaries were evaluated, while deep cortical and medullary circulations were not assessed. Given the complexity of the renal architecture corresponding to different vascular organizations, it should be acknowledged that our experiments only target a very limited spectrum of the entire span of renal capillaries. Finally, variabilities in RBF and Vm measurements were lowered by sepsis, such as the model better predicted individual values for a given MAP. This is in keeping with previous observations of a loss of spontaneous heart rate variability in patients with multiple organ dysfunction or sepsis [17]. A blunted autonomic function has been hypothesized in sepsis to explain these data, however the relevance of this altered variability for organ dysfunction remains unknown.

Several limitations of our study have already been discussed above. Notably, in this 'kidney-centered' study, parameters (including cardiac output, renal venous pressure, renal interstitial pressure, inflammation parameters, and so on) with potential interest in deciphering the relationship observed between MAP and RBF could not be measured. However, this study is nonetheless relevant with regard to one of the more prevailing questions in clinical practice for patients in shock conditions, namely the MAP target that should be reached through fluid loading and NE. We were not able to measure renal filtration during our MAP steps due to limited time spent at each step. Our evaluation of renal function with serum creatinine at the end of the experiments represents the sum of all insults that occurred to the kidney throughout each experiment. It is thus impossible to discriminate precisely at which time point changes in glomerular filtration occurred and eventually became permanent, which limits what can be inferred from creatinine measurements. At least, the observation that sepsis led to increased serum creatinine confirms the relevance of our model.

## Conclusions

Our resuscitated rat model adds to the knowledge on renal macro-hemodynamics in sepsis in that the ability of the kidney to protect itself by maintaining RBF when MAP varies above a certain MAP threshold does not appear to be altered by the presence of sepsis or NE infusion. Notwithstanding, a direct vasoconstrictive effect of NE was evidenced. In contrast with the absence of any observable effect of sepsis on the relationship between MAP and RBF in resuscitated animals, from a microcirculatory standpoint, sepsis is very rapidly associated with 'hypervelocity' of blood flow in cortical peritubular capillaries, which is reversed by NE infusion. The mechanisms and consequences of this discrepant effect of sepsis on renal macro and microcirculations may be of great importance in understanding the failure of glomerular function in sepsis.

## Key messages

• In a rat model, early sepsis does not modify the relationship between renal blood flow and mean arterial pressure.

• Conversely, a vasoconstrictive effect of norepinephrine was observed.

• Sidestream Dark Field imaging enabled to observe blood hypervelocity in cortical peritubular capillaries in sepsis, which was improved by norepinephrine

• Thus, sepsis appears to differently impact kidney macro- and microcirculations

## Abbreviations

AKI: acute kidney injury; CBF: carotid blood flow; CT group: control group; CT+NE group: control + norepinephrine group; GFR: glomerular filtration rate; HR: heart rate; MAP: mean arterial pressure; NE: norepinephrine; RBF: renal blood flow; Sepsis+NE group: sepsis + norepinephrine group; SDF: Sidestream Dark Field; Vm : cortical microcirculatory velocity.

## Competing interests

The authors declare that they have no competing interests.

## Authors' contributions

MB participated in the design of the study, acquired the data, and drafted the manuscript; JFH participated in the design of the study and performed the statistical analysis; MT participated in the design of the study, acquired the data, and drafted the manuscript; MdB participated in the design of the study, acquired the data, and drafted the manuscript; AD participated in the design of the study, acquired the data, and drafted the manuscript; AM interpreted the data and drafted the manuscript; PC and AM interpreted the data and drafted the manuscript; PA and NL conceived and designed the study, interpreted the data and drafted the manuscript. All authors read and approved the final manuscript.

## Supplementary Material

Additional file 1 **Video S1 (online)**. **Video sequences of the kidney surface obtained with a Sidestream Dark Field (SDF) camera**. After image stabilization and averaging, displacement of blood cells in cortical peritubular capillaries can be observed. Artifacts created by displacement of white blood cells among red blood cells can be readily observed and measured. Click here for file

Additional file 2 Click here for file
