# Supplementary material for: Renal macro- and microcirculation autoregulatory capacity during early sepsis and norepinephrine infusion in rats
Source: Crit Care. 2013 Jul 12;17(4):R139. doi: 10.1186/cc12818 (PMC4056525; doi:10.1186/cc12818)
Supplement: Additional file 2 [file cc12818-S2.DOCX]

**Table S1 :** Numerical data of measured variables in the experimental groups.

|  | **Control (n=10)** | **Sepsis (n=10)** | **Norepinephrine (n=10)** | **Sepsis+**  **Norepinephrine (n=10)** |
| --- | --- | --- | --- | --- |
| **Mean Arterial Pressure (mmHg)** |  |  |  |  |
| Initial | 129±13.2 | 130±3.5 | 133±9.8 | 129±2.7 |
| Initial Norepinephrine |  |  | 160±6,8 | 160±2.4 |
| Level 130 mmHg |  |  | 131±6,9 | 130±3.6 |
| Level 100 mmHg | 98±13.1 | 101±3.6 | 101±6.9 | 100±3.1 |
| Level 70 mmHg | 68±12.8 | 70±3.4 | 70±6.3 | 69±2.7 |
| Level 40 mmHg | 47±7.7 | 40±3.1 | 41±6.6 | 40±2.4 |
| Reperfusion | 127±26.0 | 100±35.2 | 109±40.9 | 119±29.6 |
| **Heart Rate (beats/min)** |  |  |  |  |
| Initial | 324±63.1 | 427±51.0 | 342±45.2 | 371±66.1 |
| Initial Norepinephrine |  |  | 405±49.5 | 442±84*.*5 |
| Level 130 mmHg |  |  | 429±43.9 | 457±86.8 |
| Level 100 mmHg | 315±53.6 | 420±54.8 | 441±39.5 | 473±84.4 |
| Level 70 mmHg | 290±49.1 | 402±52.1 | 442±45.2 | 480±71.6 |
| Level 40 mmHg | 332±75.9 | 417±63.5 | 441±34.1 | 446±49.1 |
| Reperfusion | 354±79.7 | 447±62.9 | 426±31.0 | 406±62.0 |
| **Carotid Blood Flow (mL/min)** |  |  |  |  |
| Initial | 3.3±2.9 | 4.1±2.3 | 1.6±0.8 | 2.1±0.7 |
| Initial Norepinephrine |  |  | 2.4±0.9 | 2.6±0.9 |
| Level 130 mmHg |  |  | 1.3±0.4 | 1.8±0.9 |
| Level 100 mmHg | 1.7±1.9 | 2.4±1.4 | 0.8±0.3 | 1.8±0.9 |
| Level 70 mmHg | 1.4±1.5 | 1.9±1.1 | 0.8±0.5 | 1.4±0.5 |
| Level 40 mmHg | 0.8±0.7 | 1.1±0.5 | 0.6±0.4 | 1.0±0.6 |
| Reperfusion | 2.9±2.9 | 1.8±1.4 | 1.6±0.9 | 2.3±0.9 |
| **Left Renal Blood Flow (mL/min)** |  |  |  |  |
| Initial | 3.2±3.4 | 2.5±2.0 | 1.8±0.9 | 3.4±1.2 |
| Initial Norepinephrine |  |  | 1.4±0.8 | 1.6±0.9 |
| Level 130 mmHg |  |  | 1.1±0.3 | 1.3±0.7 |
| Level 100 mmHg | 2.5±2.4 | 1.9±1.5 | 1.1±0.3 | 1.3±0.7 |
| Level 70 mmHg | 1.4±1.2 | 1.2±0.7 | 1.1±0.6 | 1.0±0.5 |
| Level 40 mmHg | 0.7±0.6 | 0.5±0.4 | 0.9±0.4 | 0.7±0.3 |
| Reperfusion | 2.1±1.9 | 1.3±0.8 | 1.2±0.5 | 1.2±0.4 |
| **Microcirculatory Velocity (Vm, µL/min)** |  |  |  |  |
| Initial | 305.3±62.4 | 387.1±135.0 | 424.1±60.5 | 456.2±71.9 |
| Initial Norepinephrine |  |  | 374.1±95.8 | 388±84.1 |
| Level 130 mmHg |  |  | 302.7±94.9 | 307.8±43.6 |
| Level 100 mmHg | 316.7±74.7 | 339.1±131.8 | 288.4±82.9 | 277.3±82.2 |
| Level 70 mmHg | 262.8±67.4 | 408.4±107.3 | 239.7±35.7 | 305.4±45.4 |
| Level 40 mmHg | 193.4±47.8 | 239.9±75.9 | 210.4±32.8 | 226.0±36.9 |
| Reperfusion | 332.0±80.5 | 379.0±126.8 | 401.0±91.7 | 384.4±79.6 |
